# Challenges of Retrograde Ureteroscopic Procedures in Overweight Patients

**DOI:** 10.7759/cureus.47815

**Published:** 2023-10-27

**Authors:** Abdolsalam Ahmadi, Ahmed A Al Rashed, Omran Hasan, Batool M Turki, Ali H Al Aradi, Khalid Abdulaziz, Nader Awad, Akbar Jalal

**Affiliations:** 1 Urology, Salmaniya Medical Complex, Manama, BHR; 2 Urology, Salmaniya Medical Complex, Manama , BHR

**Keywords:** ureteroscopic lithotripsy, urolithiasis epidemiology, treatment of urolithiasis, obesity and nutrition, semirigid ureteroscope

## Abstract

Background: Obesity and urolithiasis are both prevalent conditions that have an impact on the healthcare system. The ureteric diameter and accessibility play a crucial role in the management of urolithiasis in both overweight and normal weight patients. Studies have shown that obesity can lead to changes in ureter diameter where excessive body fat can exert pressure on the kidneys, causing them to enlarge in size and this enlargement can result in a compression of the adjacent structures, including the ureter. The aim of this study is to assess the incidence of intraoperative challenges faced during retrograde ureteroscopic procedures in overweight patients with ureteric and renal calculi.

Methods: We retrospectively reviewed patients who underwent retrograde ureteroscopic surgery (RURS) for urolithiasis from 1^st^ January 2021 until 30^th^ August 2023. The outcome and any complications were documented and compared with the patient’s Body Mass Index (BMI). All patients who undergo RURS in our facility have to have a Non-Contrast CT scan prior to surgery. Procedural success was determined by the ability to obtain access to the stone site intraoperatively and stone-free status in kidney, ureter, and bladder (KUB) X-ray post-operatively. Post-operative complications were recorded up to two weeks post-operatively and classified according to the Calvein Dindo Classification.

Results: Our total sample size was 146 patients out of which 75 were overweight and 71 were normal weight patients. In 34 (45%) of overweight patients’ access to the ureter was restricted due to a narrow ureteric orifice with ureteroscopy not successful; on the contrary 13 (18%) of normal weight patients faced this same issue. This was statistically significant with a p-value of .004. The stone clearance rates were 91% and 95% in overweight and normal weight patients respectively, which is higher in normal weight patients however this difference was not found to be a statistically significant finding (p-value .028). Overweight patients had 12% Grade I and 8% Grade II complications whereas normal weight patients had 11% Grade I complications and 1.4% Grade II with no higher-grade complications.

Conclusion: Retrograde ureteroscopic procedures are a safe treatment modality for patients with urolithiasis in both overweight and normal weight populations. They are shown to have similar success rates between both populations once ureteric access is obtained. However, access failure rates are shown to be slightly higher in overweight patients. Hence, further preoperative patient counselling and technical considerations should be undertaken.

## Introduction

Obesity has been a prevalent health issue in recent years which is characterized by excessive body fat accumulation and which has proven to have numerous negative effects on one's overall health [1]. Apart from the well-known implications such as endocrine disorders, cardiovascular diseases and joint problems, obesity can also have an impact on the genitourinary system which can lead to various technical difficulties encountered intraoperatively.

Urolithiasis is also a prevalent condition worldwide causing almost 1.1 million emergency department visits in the United States with a primary diagnosis of renal calculus or colic [2]. The ureter is a structure connecting the kidneys to the urinary bladder and its primary function is to facilitate the passage of urinary flow into the bladder [3]. Hence, the diameter of the ureter will have a direct impact on the rate of urinary flow [3]. A narrower ureter can have several implications for an individual's urinary system. Firstly, it can hinder the flow of urine from the kidneys to the bladder, potentially leading to urinary stasis [4]. The sequelae of urinary stagnation include an increase in the risk of urinary tract infection and stone formation [4]. Additionally, a narrower diameter can result in difficulty accessing the ureter during retrograde ureteric procedures hence increasing the incidence of procedural complications [4].

There are several factors that can contribute to the development of a narrow ureter. These include congenital abnormalities present at birth as well as acquired conditions like renal and ureteric calculi, tumors, or even scarring and fibrosis due to previous surgeries [4]. However, studies have shown that obesity can lead to changes in ureter diameter where excessive body fat can exert pressure on the kidneys, causing them to enlarge in size and this enlargement can result in a compression of the adjacent structures, including the ureter [4].

There are multiple clinical sequelae that surgeons may encounter when performing ureteric procedures on patients with narrow ureters. These include difficulty in accessing the ureter, which can prolong surgery and increase the risk of injury to surrounding structures such as the urinary bladder [5]. Furthermore, these cases have an increased risk of ureteral injury intraoperatively such as ureteral perforation, leakage, or strictures, which may require additional procedures for repair [5].

The aim of this study is to assess the incidence of intraoperative challenges faced during retrograde ureteroscopic procedures in overweight patients with ureteric and renal calculi.

## Materials and methods

We retrospectively reviewed patients who underwent retrograde ureteroscopic surgery (RURS) for ureteric urolithiasis from 1^st ^January 2021 until 30^th ^August 2023 in Salmaniya Medical Complex, which is the major healthcare facility in the Kingdom of Bahrain. The outcome and any complications that occurred were documented and compared with the patient’s Body Mass Index (BMI). BMI classification was done according to the World Health Organization (WHO) guidelines in which a BMI of 18.5 to 25 kg/m^2^ is considered normal, overweight is established by a BMI of 25-29.9 kg/m^2^, obesity is established at a BMI ≥ 30 kg/m^2 ^and morbid obesity is present at a BMI ≥ 40 kg/m^2 ^[6].

Moreover, exclusion criteria included patients already on Double J (DJ) stents, patients with known congenital malformations of the ureter and patients with malignancy involving the ureteric orifices or the intramural part of the ureter. All patients who undergo RURS in our facility have to have a Non-Contrast CT scan (NCCT) prior to surgery.

All procedures were conducted in the same operating room and with the same semi-rigid Karl Storz 6/7Fr ureteroscope (Karl Storz SE & Co KG, Tuttlingen, Germany). A Bard Inlay 4.6 Fr 24-26cm DJ stent (C.R. Bard, Inc., New Jersey, USA) was deployed when required. Cases that required a DJ stent were mainly due to failure of access to the ureter using a semi-rigid ureteroscope or if an unhealthy-appearing ureter was seen post-procedure. If deployed, the DJ stent was kept in place for six weeks duration. Additionally, any lithotripsy that was required was done using JenaMed Holmium Laser (JenaSurgical GmbH, Jena, Germany) with pulse energy settings ranging between 0.6 and 1 J and frequency of 10 and 20 Hz.

The standard technique for ureteroscopic surgery in our facility in the treatment of urolithiasis starts with a 19 Fr 30-degree cystoscope followed by a retrograde pyelogram and then the placement of a 0.003-inch safety wire (sensor and hydrophilic wires were deployed when necessary) to maintain access followed by the ureteroscopy and laser lithotripsy if feasible. Basket retrieval of stone fragments was conducted when necessary and all procedures were performed under general anesthesia. Procedural success was determined by the ability to obtain access to the stone site intraoperatively and stone-free status in kidney, ureter and bladder (KUB) X-ray post-operatively. Any stone fragments larger than 2 mm on the post-operative X-ray KUB were regarded as a failure of complete stone clearance.

Post-operative complications were recorded up to two weeks post-operatively and classified according to the Calvein Dindo Classification [7]. Grade I complications included patients who developed post-operative urinary tract infections (UTIs), required strong opioid analgesics, or prolonged indwelling Foley catheter to allow for irrigation of gross hematuria. Grade II complications included patients who developed post-operative atelectasis, Lower respiratory tract infections, or severe hematuria needing a blood transfusion. Grade III was considered in patients who required radiological or surgical intervention. Grade 4 was considered for patients who developed life-threatening conditions and finally, grade 5 was reserved for patients who died.

All statistical analysis was conducted using SPSS software version 29.0 (IBM Corp., Armonk, NY) and 95% confidence intervals were calculated for the treatment’s success rates with p-values of < 0.005 considered statistically significant.

## Results

A total of 146 retrograde ureteroscopies were performed under the inclusion criteria during the timeframe of the study. From our total sample, 75 patients were considered overweight and higher (BMI ≥ 25 kg/m^2^) with an average age of 41.6 years. Their average weight was 104.3 kg with a mean BMI of 38.8 kg/m^2^. The overall average individual stone size was 7.8 mm. Conversely, 75 patients were considered obese (BMI ≥ 30 kg/m^2^) with an average age of 41.6 years. Their average weight was 104.3 kg with a mean BMI of 38.8 kg/m^2^. The overall average individual stone size was 7.8 mm. On the other hand, 71 patients in our study were of normal weight (BMI 18.5 to 25 kg/m^2^) with an average age of 38.2 years. The mean BMI was 23.8 kg/m^2^ and the mean individual stone size was 8.3 mm.

**Figure 1:**
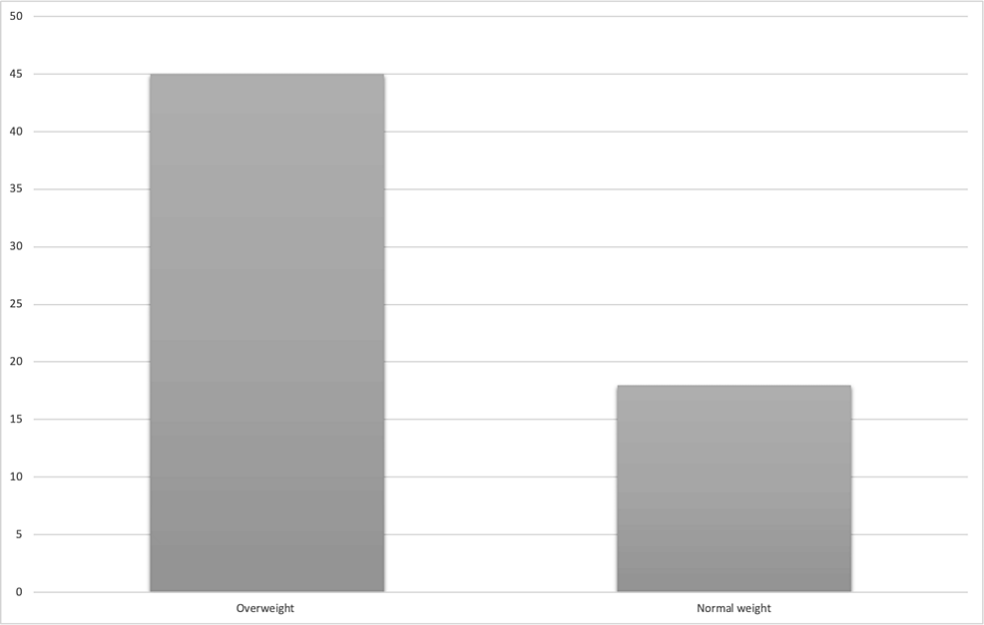
Percentages of procedures using Inlay DJ stent Percentages of procedures in overweight versus normal weight patients in which access to the ureter with a semi-rigid 6/7 Fr Karl Storz Ureteroscopy was not possible and Inlay DJ stent was kept in place.

Furthermore, in 34 (45%) of the overweight patients’ sample, access to the ureter was restricted due to a narrow ureteric orifice or intramural part with ureteroscopy not successful which required aborting the procedure and insertion of 4.6 Fr DJ stent followed by a second session once ureter was dilated by the stent. On the contrary, 13 (18%) of normal weight patients faced this same issue, which was statistically significant with a p-value of .004. These findings can be illustrated in Figure 1 below.

Moreover, stone clearance rates were measured in all patients with successful ureteroscopic access and lithotripsy from the first procedure. This was done by assessing a KUB X-ray 24 hours post operatively. Although the mean individual stone burden was slightly higher in normal weight patients (8.2 mm vs 7.8 mm), this was not a statistically significant finding (p-value 0.805). The stone clearance rates were 91% and 95% in overweight and normal weight patients, which is higher in normal weight patients however this difference was not found to be a statistically significant finding (p-value .028).

Overweight patients had 12% Grade I and 8% Grade II complications and no higher-grade complications were recorded. Normal weight patients had 11% Grade I complications and 1.4% Grade II with no higher-grade complications. These findings showed no statistical significance in grade I complications (p-value 0.490) but a statistically significant increase in overweight patients over normal weight in Grade II complications (p-value 0.003). These findings can be better illustrated in Table 1 below.

**Table 1:** Calvien Dindo complication grades in normal weight versus overweight patients within two week post-operative period

Calvien Dindo Grade	Normal weight patients	Overweight patients
Grade I	8/71 (11%)	9/75 (12%)
Grade II	1/71 (1.4%)	6/75 (8%)
Grade III	Nil	
Grade IV		
Grade V		

## Discussion

Overweight patients present multiple challenges for urologists worldwide. These challenges range from urolithiasis symptoms presenting more vaguely with the pain being more generalized, abdominal fat making the physical examination more difficult and even imaging accuracy being diminished from poor image quality [8]. The plain X-ray films become scattered and during renal ultrasound, the kidney’s depth makes them hard to localize [8]. 

Furthermore, our results demonstrate two statistically significant issues as they relate to overweight patients and retrograde ureteroscopic procedures. The first is the higher ureter access failure rate in overweight patients as compared to normal weight (45% versus 18%). This creates an added burden on the healthcare system and the patient as these patients will require another scheduled operation while in the meantime will need to be placed on a DJ stent. In addition to the financial cost of an added procedure, the stents cause significant morbidity to patients as they are associated with flank and abdominal pain, hematuria, urgency with urge incontinence, and recurrent UTIs [8].

In terms of stone-free clearance for patients in which ureteric access was feasible, our data suggests that there is no statistically significant difference between the two sets of patients. These findings are similar to a study conducted in 2012, which demonstrated that the stone-free rate for the ureter was 91% in obese patients and 95.4% in normal weight cases [9].

Secondly, our results showed a statistically significant increase in grade II Calvien Dindo complications amongst overweight patients however the complications observed were mostly unrelated to the ureteroscopic procedure but rather related to the morbidity of obesity due to anesthesia, decreased preoperative cardiac function and increased susceptibility to infections due to worsening respiratory function and decreased mobility [10].

Moreover, the treatment for urolithiasis varies in scope according to stone size and location. RURS remains a common and safe method of surgical approach however there has been an increasing trend in the use of Extracorporeal Shockwave Lithotripsy (ESWL) due to its non-invasive approach coupled with the avoidance of anesthesia [11]. Currently, ESWL is the recommended approach for ureteric stones smaller than 1 cm and visible on a KUB X-ray and the procedure has a stone-free clearance rate reaching up to 92.6% for proximal stones and 97.5% for distal stones [11]. However, although our data showed retrograde ureteroscopic access is less successful in overweight patients, the use of ESWL in some cases is contraindicated. Such cases include obese patients whose weight exceeds that of the ESWL table capacity and in which subcutaneous fat increases the skin to stone depth which prevents successful lithotripsy using the shockwaves [11]. This demonstrates that ESWL is much more dependent on the patient’s body habitus and weight than RURS. Furthermore, Muñoz et al. found a 72% stone-free rate in ESWL obese patients [12], which is lower than the RURS stone-free clearance rate seen in our overweight patients which was 91%. Consequently, ESWL may represent a sub-optimal approach for obese patients and a RURS approach can be considered a valid alternative.

Another common approach for urolithiasis is Percutaneous Nephrolithotomy (PCNL), which is particularly used in patients whose stone burden is larger than 2 cm or between 1-2 cm and present in the lower pole of the kidney [13]. This is due to the fact that PCNL will provide a fast and safe approach for the removal of these larger stone burdens in a single setting. However, PCNL in overweight patients creates multiple challenges for urologists such as patient positioning, imaging for access, longer skin-to-collecting-system distances, and nephrostomy tube dislodgement [14]. In these cases, PCNL is not feasible and even carries a higher risk of complications and surgeons might opt for multiple flexible ureteroscopic procedures over PCNL [13-14].

As the understanding of the technical aspects of retrograde ureteric access in RURS procedures improves, we are able to innovate in ways that make such procedures safer for patients. Advancements have been made by equipping semi-rigid ureteroscopes with a flexible distal tip to ease access into tight or tortuous ureters. As these ideas are realized and practiced more in clinical settings, the traditional semi-rigid ureteroscope can potentially be equipped with several mechanisms that aid the surgeon to navigate tight ureters including potentially adding a pneumatic balloon to the distal tip of the scope which can inflate when needed. This can provide the required dilation and thus facilitate easier and safer access into tight ureters.

These technical challenges highlight how ureteroscopic procedures are an invaluable tool in the management of urolithiasis particularly in obese and overweight patients. Further understanding of the complications that may affect the success rates of RURS procedures in this population should be well understood and improved wherever possible.

Finally, limitations of our study include not being able to perform a post-operative non-contrast CT KUB for post-operative stone clearance rates measurement. This was not done to avoid higher financial costs and to reduce the radiation exposure to patients. Another limitation of this study is that the procedures were conducted with different urologists with different experience levels which can impact success rates.

## Conclusions

Retrograde ureteroscopic procedures are a safe treatment modality for patients with urolithiasis in both overweight and normal weight populations. They are shown to have similar success rates between both populations once ureteric access is obtained. However, access failure rates are slightly higher in overweight patients. Hence, further preoperative patient counselling and technical considerations should be undertaken and possible future innovations can be done to assist in ureteric access in such populations.
